# Transcriptomic Profiling of Influenza A Virus-Infected Mouse Lung at Recovery Stage Using RNA Sequencing

**DOI:** 10.3390/v15112198

**Published:** 2023-10-31

**Authors:** Huda A. M. Al-Shalan, Dailun Hu, Penghao Wang, Jasim Uddin, Abha Chopra, Wayne K. Greene, Bin Ma

**Affiliations:** 1 School of Medical, Molecular and Forensic Sciences, Murdoch University, Murdoch, WA 6149, Australia; mic_humu2012@covm.uobaghdad.edu.iq (H.A.M.A.-S.); p.wang@murdoch.edu.au (P.W.); jasim.uddin@murdoch.edu.au (J.U.); w.greene@murdoch.edu.au (W.K.G.); 2 Department of Microbiology/Virology, College of Veterinary Medicine, Baghdad University, Baghdad 10071, Iraq; 3 Department of Pathogenic Biology, Hebei Medical University, Shijiazhuang 050017, China; 17300574@hebmu.edu.cn; 4 Genomics Core Research Facility, Health Futures Institute, Murdoch University, Murdoch, WA 6149, Australia; a.chopra@iiid.murdoch.edu.au

**Keywords:** transcriptomic analysis, influenza A virus, RNA sequencing, respiratory infection, neuroregulation, immunometabolism

## Abstract

Influenza A virus (IAV) is known to cause mild to severe respiratory illness. Under some conditions, the infection can lead to pneumonia (viral or bacterial), acute respiratory distress syndrome, and other complications that can be fatal, especially in vulnerable populations such as the elderly, young children, and individuals with underlying health conditions. Despite previous studies, little is known about the host immune response and neuroimmune interactions in IAV infection. Using RNA sequencing, we performed transcriptomic analysis of murine lung tissue 21 days post infection (dpi) with IAV (H1N1) in order to find the differentially expression genes (DEGs) related to the host immune response and neuroimmune interactions inside the lung during recovery. Among 792 DEGs, 434 genes were up-regulated, whereas 358 genes were down-regulated. The most prominent molecular functions of the up-regulated genes were related to the immune response and tissue repair, whereas a large proportion of the down-regulated genes were associated with neural functions. Although further molecular/functional studies need to be performed for these DEGs, our results facilitate the understanding of the host response (from innate immunity to adaptive immunity) and neuroimmune interactions in infected lungs at the recovery stage of IAV infection. These genes might have potential uses as mechanistic/diagnostic biomarkers and represent possible targets for anti-IAV therapies.

## 1. Introduction

Influenza A virus (IAV) is a highly infectious respiratory pathogen that belongs to the Orthomyxoviridae family [1,2]. It is known to cause seasonal epidemics and occasional pandemics that pose a significant threat to public health worldwide [1,2,3]. The virus is characterized by its segmented genome and its ability to undergo rapid antigenic evolution, which allows it to evade the host immune system and to establish new infections. IAV is known to cause mild to severe respiratory illness and symptoms can include fever, cough, sore throat, fatigue, and muscle aches. Under some conditions, the infection can lead to pneumonia (viral or bacterial), acute respiratory distress syndrome (ARDS), and other complications that can be fatal, especially in vulnerable populations such as the elderly, young children, and individuals with underlying health conditions [1,2]. The H1N1 (A(H1N1)pdm09) influenza pandemic of 2009 (swine flu pandemic) was a global outbreak of the H1N1 IAV in this century. It started in Mexico and quickly spread to other countries, causing widespread public health concerns [4]. According to the data from the World Health Organization (WHO), the annual epidemics of IAV are estimated to cause about 3 to 5 million cases of severe illness and about 290,000 to 650,000 respiratory deaths worldwide.

The clinical manifestations of IAV infections are mediated by the interplay/crosstalk between the virus and the host immune system [5]. Upon infection, the virus replicates first in the respiratory tract, leading to the activation of innate immune responses and the recruitment of immune cells to the site of infection. The virus can also modulate host immune responses by suppressing the production of interferons and other cytokines that are critical for antiviral defense. The A(H1N1)pdm09 strain can induce higher levels of inflammatory cytokines, which make it more virulent compared with other strains [6].

During viral infection, alterations of viral and host gene expression have been identified by using methods such as next-generation sequencing (NGS) [7]. For instance, IAV infection is known to induce profound changes in the host transcriptome [8] and reveals definite sets of gene expression phenotypes including RNA polymerase readthrough [9,10,11], translational shutoff [12], altered genome architecture [10], and transcript degradation [13]. However, the pathogenesis of H1N1 infection at the gene expression level, specifically during the recovery period of infection, has not been elucidated.

Previously, the analysis of whole-genome gene expression has been achieved through the use of microarray platforms such as the Affymetrix Gene Chip system [14]. Recently, RNA sequencing (RNA-seq) has been utilized as an effective tool to assess the differential gene expression of all types of RNAs, including messenger RNAs (mRNAs), transfer RNAs (tRNAs), ribosomal RNAs (rRNAs), non-coding RNAs (ncRNAs), small RNAs (sRNAs), and long non-coding RNAs (lncRNAs) [15]. It is an efficient quantitative method capable of capturing the expression of almost all transcripts expressed in a genome [16].

Since our particular interests are the neuroimmune crosstalk/interactions in health and diseases [17,18], our aim here was to study the molecular basis of neuroimmune relationships throughout IAV infection, in addition to exploring novel differentially expressed genes (DEGs) related to the host immune response. Despite previous studies on the short-term effect of IAV infection [19], the host immune response and neuroimmune interactions in the recovery stage of IAV infection remain unclear. By using RNA-seq, we performed transcriptomic analysis of murine lung tissue 21 days post infection (dpi) with IAV (H1N1) in order to find DEGs related to the host immune response and neuroimmune interactions inside the lungs during the recovery stage of influenza.

## 2. Materials and Methods

All animal experiments were carried out according to the recommendations of the National Health and Medical Research Council of Australia in its Guidelines to Promote the Wellbeing of Animals used for Scientific Research. All experimental procedures were approved by the Animal Experimental Ethics Committee of the Harry Perkins Institute of Medical Research (permit number: AE189).

### 2.1. Virus

The mouse-adapted human IAV H1N1 PR8 (Influenza A/Puerto Rico/8/1934, H1N1; obtained from the American Type Tissue Culture Collection) was prepared as previously outlined [20]. The virus was collected from the allantoic fluid of 10-day-embryonated chicken eggs after inoculation. The stock virus was then subcultured in Madin–Darby canine kidney (MDCK) cells (Sigma, St. Louis, MO, USA) by using Dulbecco’s modified Eagle’s medium (DMEM; Gibco, Sydney, Australia) and harvested as the tissue culture supernatant. To prepare a confluent monolayer of MDCK cells, 1.0 × 10^4^ cells (in 100 µL growth media) were seeded in each well of 96-well plates. After a series of diluted virus samples (10^−2^ to 10^−7^) were produced from the original virus sample, 100 µL diluted sample was added to each well in 96-well plates (Thermo Fischer Scientific, Waltham, MA, USA). The viral titers were determined by observing the cytopathic effects on MDCK cells (under the microscope without staining) over a 5-day incubation period (at 37 °C with 5% CO_2_). The viral titer was calculated using the method of Muench and Reed and expressed as the mean log 10 tissue culture infective dose that killed 50% of the cells (TCID_50_).

### 2.2. Mouse Infection and Lung Sample Collection

Eight 7-week-old female C57BL/J6 mice were purchased from the Animal Resource Centre (Perth, WA, Australia). All mice were kept in individually ventilated filter cages with autoclaved food under specific pathogen-free conditions with a 12 h dark/light cycle (room temperature: 25 °C; relative humidity: 60%). They were randomly divided into the control group (3 mice) and the infection group (5 mice).

The mice were inoculated intranasally (i.n.) under light inhalation anesthesia (with isoflurane; Sigma) with 20 TCID_50_ H1N1 diluted in 50 µL sterile phosphate-buffered saline (PBS; Sigma). For the control group, only 50 µL PBS was used. Fresh lung tissue samples from 3 non-infected and 5 infected mice at the recovery stage of experimental IAV infection (21 dpi) were examined. The lung tissues were collected, immediately stored in RNALater solution (Thermo Fischer Scientific; in order to preserve RNA integrity), and kept at −80 °C until further processing and analysis.

### 2.3. RNA Extraction and Purification

Total RNA was extracted from lung tissues preserved in RNALater solution by using Trizol reagent (Thermo Fischer Scientific). The aqueous phase of the Trizol extract was purified with the PureLink^TM^ RNA Mini Kit (Thermo Fischer Scientific) following the manufacturer’s instructions.

The first step involved homogenization of the lung tissue. Every tissue sample collected was cut into small pieces and weighed. On average, each sample weighed between 20 and 40 mg, the heaviest samples reaching 100 mg in weight. After being weighed, samples were then placed inside a sterile ribonuclease (RNase)-free polypropylene tube (Thermo Fischer Scientific). An appropriate amount of Trizol reagent (Sigma) was added to each tube according to the weight of each tissue sample. A volume of 800 μL was added for smaller samples (1–40 mg), and 1 mL reagent was used for larger samples (41–100 mg). The samples were then homogenized with a rotor–stator homogenizer (Heidolph DIAX 600; Heidolph Instruments, Schwabach, Germany). The rotator was set to mix at a rate of 2400 rpm. Subsequently, the homogenate was centrifuged at 12,000× *g* for 5 min. The supernatant was then transferred into a clean RNase-free tube.

The second step was phase separation. The supernatant in new test tubes was then incubated for 5 min at 15 °C to 30 °C to permit the complete dissociation of nucleoprotein complexes. After incubation, 0.2 mL chloroform (per 1 mL of Trizol reagent; Sigma) was added to the samples, and the tubes were then capped, shaken vigorously by hand for 15 s, and incubated again for 2–3 min at 15–30 °C. Following this incubation step, samples were transferred into a Phasemaker™ tube and centrifuged at 12,000× *g* for 15 min at 4 °C resulting in a notable aqueous layer, which was extracted and transferred into a clean RNase-free tube.

The third step led to the binding of the RNA, which was then washed. Ethanol (100%) was added to the aqueous solutions until the ethanol concentration reached 35%. After being mixed by vortexing briefly, samples were spun for a short time on a capsulefuge. Up to 700 μL of the sample (including any remaining precipitate) was transferred to the spin cartridge (with the collection tube; both were from the PureLink^TM^ RNA Mini Kit) according to the manufacturer’s instructions. The collection tubes (with the spin cartridge inside) were then centrifuged at 12,000× *g* for 30 s at room temperature. The flow-through into the collection tube was discarded together with the collection tube. Subsequently, 700 μL wash buffer I solution was added to the cartridge (with a new collection tube). The collection tubes (with the spin cartridge inside) were then centrifuged at 12,000× *g* for 30 s at room temperature. The flow-through into the collection tube was discarded together with the collection tube again. The spin cartridge was placed into a new collection tube, and the washing step was repeated once. Next, 700 μL wash buffer II work solution (containing 140 μL wash buffer II and 560 μL ethanol) was then added to the cartridge (with a new collection tube). The collection tubes (with spin cartridge inside) were then centrifuged at 12,000× *g* for 15 s at room temperature. The flow-through into the collection tube was discarded together with the collection tube. The spin cartridge was transferred into a new collection tube, and the washing step was repeated once.

The fourth step was the elution of the RNA. The spin cartridge with a new collection tube was centrifuged at 12,000× *g* for 2 min. The collection tube was then discarded, and the spin cartridge was inserted into a recovery tube to collect the RNA. RNase-free water (50 μL) was directly added to the center of the spin cartridge, and the recovery tube (with spin cartridge) was incubated for 1 min at 25–28 °C and then centrifuged at 12,000× *g* for 2 min. The RNAs eluting from the cartridge were subsequently collected in the recovery tube. The quantity and quality of the extracted RNA were assessed by usingNanodrop spectrophotometry (Wilmington, DE, USA), denaturing agarose gel electrophoresis, and an Agilent 2100 Bioanalyzer (Agilent Technologies, Mulgrave, VIC, Australia).

### 2.4. Library Preparation and RNA-Seq

The RNA-seq of all the experimental samples was performed at the Genomic Core Research Facility, Health Futures Institute, Murdoch University.

The total RNA was diluted with RNase-free water to 100 ng/µL, and 500 ng RNA was used with tagged primers for the production of cDNA (by using an adapted Smart-Seq assay targeting the poly-A tail of mRNAs) [17]. Conventionally, RNA was hybridized to oligo(dT)-containing primers (/5Biosg/AAG CAG TGG TAT CAA CGC AGA CA TTTAGG (N:25252525)(N)(N)(N)(N)(N)(N)(N) 30T*V*(N)). The first strand of the cDNA was synthesized with the addition of a few untemplated C nucleotides. This poly (C) overhang was exclusively added to full-length transcripts. An oligonucleotide primer was hybridized to the poly (C) overhang and used to synthesize the second strand. The sequence for template switching oligo (TSO) for generating the double-stranded product was AAGCAGTGGTATCAACGCAGAGTACATrGrG + G. The full-length cDNAs were amplified, purified, and quantified using the Promega Quantus Fluorometer (Promega, Alexandria, NSW, Australia).

The NEBNext^®^ Ultra™ II FS DNA Library Prep Kit for Illumina (New England Biolabs; Ipswich, MA, USA)/Illumina DNA Prep (Illumina; San Diego, CA, USA) was then used to prepare the double-stranded cDNA library for RNA-Seq on the Novaseq 6000 (Illumina; using 100 ng of input double-stranded cDNA) according to the manufacturer’s instructions.

All the raw RNA-Seq reads were analyzed by means of FastQC (version 0.10.1; https://www.bioinformatics.babraham.ac.uk/projects/fastqc/; accessed on 15 December 2020) and in-house software Biokanga (version 3.6.3) to check for read quality, as was performed using the newest Illumina protocol.

### 2.5. Sequence Alignment and Gene Annotation

High-quality reads were independently aligned, by using the Spliced Transcripts Alignment to a Reference (STAR) aligner (version 2.5.3a) [18], against the *Mus* musculus (strain C57BL/6J) complete DNA assembly reference sequence and annotation obtained from the National Center for Biotechnology Information (NCBI). Alignment parameters were set with a maximum of two mismatches being allowed. Mismatches of two were used to accommodate potential base-calling errors. Only uniquely aligned reads were retained for analysis, with reads that aligned to multiple loci being discarded. A count matrix was generated and loaded into R (version 3.5.1) for downstream statistical analysis.

For gene annotation, the RNA-seq read alignment was annotated using the official gene transfer format (GTF) annotation file obtained from the NCBI by means of in-house R and C^++^ scripts. The annotation from the NCBI for RNA-seq data contained the chromosome and loci information. Gene symbols and Ensembl gene IDs were used to match the results obtained from the RNA-seq.

### 2.6. Statistical Analysis of DEGs

The edgeR package (version 3.2.4) was used for DEG analysis [21]. Read counts were normalized by following edgeR′s model-based Trimmed Mean of M-values method, followed by the fitting of a negative binomial generalized linear model accommodating the disease type (infected mice or control mice). Metabolic pathway analysis on the DEGs was conducted using the Kyoto Encyclopedia of Genes and Genomes (KEGG) Pathways online repository (www.genome.jp/kegg/; accessed on 20 December 2020). The pathway analysis and visualization were performed in R by using the “pathfinder” package.

The transcript-wise dispersion was estimated in edgeR, allowing a possible trend with average count size. A likelihood ratio test for comparing the disease type was conducted to obtain DEGs. The resulting *p*-values were adjusted for multiple testing using Hochberg′s false discovery rate (FDR) adjustment approach [22]. DEGs were transcripts with adjusted *p*-values of less than 0.0001. The FDR cut-off of 0.05 was arbitrarily selected based on the manual inspection of the distribution of the resulting *p*-values.

The pathway analyses were performed using the PathfindR package, which utilized a novel workflow. It first performed input testing to verify any gene symbol that was not in the protein–protein interaction network (PIN) and then allocated an alias symbol if an alias was present in the PIN. Next, an active subnetwork search was performed. Pathway enrichment analyses were then carried out using the genes in each of the identified active subnetworks. Pathways with adjusted *p*-values larger than a given threshold were discarded. The lowest adjusted *p*-value (overall active subnetworks) for each pathway was kept. This process of active subnetwork search and enrichment analyses was repeated for a selected number of iterations performed in parallel. Overall iterations, the lowest/highest adjusted *p*-values, and the number of occurrences were reported for each enriched pathway.

## 3. Results

To understand the effects that IAV infection has on the transcriptomic profile of mouse lungs, 7-week-old female C57BL/J6 mice were infected with human IAV (H1N1 PR8). The changes in body weight during the infection are shown in Supplementary Figure S1. After PR8 infection, infected mice quickly lost body weight at 3–8 dpi and then gradually recovered, reaching their starting weights at 10 dpi. Between 10 dpi and 21 dpi, the body weight of both the control group and the infected group increased continuously. All mice survived at 21 dpi.

We also performed hematoxylin and eosin (H&E) and immunofluorescence staining to examine the lung histopathology at 21 dpi with PR8; the results are shown in Supplementary Figures S2 and S3. At 21 dpi, multi-foci and interstitial infiltrations of immune cells were present inside the lung. However, only a few viruses were observed inside the lung.

At 21 dpi, lung tissues from the control group (3 mice) and infection group (5 mice) were collected for mRNA-seq analysis. The total reads of the experimental samples are presented in Supplementary Figure S4, which shows good quality and consistency across all samples analyzed. An exploratory analysis was carried out on the RNA-seq data from eight lung samples (control and infected) by using principal component analysis (PCA). The transcriptional profiles of the lung tissue were distinct from each other [23].

To analyze the DEGs after infection, the edgeR package was utilized for gene differential expression analysis (*p* < 0.05). In total, 792 DEGs (434 up-regulated and 358 down-regulated) were identified (Supplementary Figure S5).

The top 100 up-regulated DEGs and their functions are shown in Table 1. These up-regulated DEGs are mainly involved in B cell/T cell/granulocyte/monocyte infiltration, leukocyte chemotaxis, innate immunity, immune modulation, inflammation/edema, complement, cell proliferation, cell signaling/apoptosis, and airway repair. Only two neuronal genes, namely calcitonin gene-related peptide-β (Calcb) and potassium voltage-gated channel subfamily A member 6 (Kcna6), were found to be up-regulated in these 100 up-regulated DEGs.

The top 100 down-regulated DEGs and their functions are shown in Table 2. These down-regulated DEGs are mainly involved in immune functions, cell division/death/adhesion, and nervous system functions. For example, the DEGs primarily involved in immune functions included genes for innate/specific immune responses, inflammatory response, and neuroinflammation. In addition, the DEGs primarily involved in nervous system functions included genes for brain development, neurotrophin signaling pathways, and neuronal activities.

By using reverse transcription polymerase chain reaction (RT-PCR), we observed the down-regulation of neurotrophin brain-derived neurotrophic factor (BDNF), glial fibrillary acidic protein (GFAP), and hemagglutinin (HA) in mouse lung during the infection (21 days) of IAV. The results are shown in Supplementary Figure S6.

**Figure 1:**
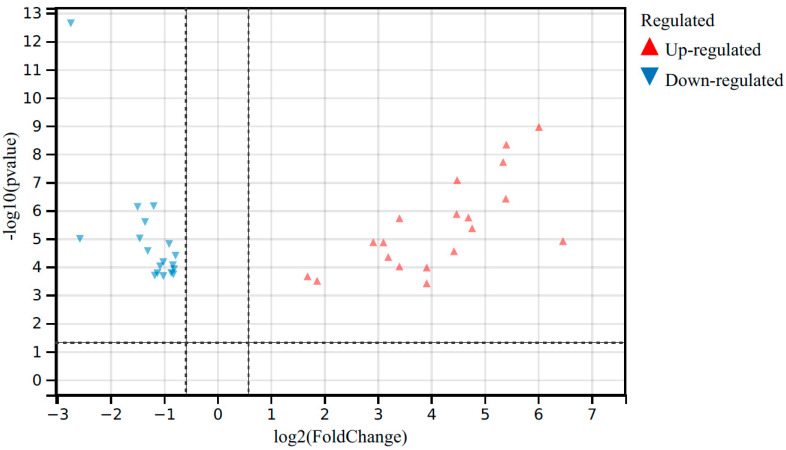
Volcano plots showing the top 20 up-regulated and 20 down-regulated DEGs in the lungs of IAV-infected mice (21 dpi) compared with control mice.

We then checked the 20 top-up-regulated and top-down-regulated DEGs in detail; the results are shown in Figure 1 and Table 3 and Table 4. One significantly up-regulated gene, which exhibited the most significant fold change in expression (162.7 times), was arachidonate 12-lipoxygenase, epidermal (ALOX12E), which is involved in the regulation of gene expression in the inflammatory response. Two other significantly up-regulated genes were keratin 14 (KRT14) and immunoglobulin kappa variable 5–48 (Igkv5–48), with fold changes of 88.9 and 64.8, respectively. KRT14 is involved in airway repair by affecting cell differentiation in epithelial tissue, whereas Igkv5–48 is a humoral immune response gene encoding the immunoglobulin light chain. Significantly down-regulated genes with the largest fold changes in expression were Spondin 2 (Spon2; 0.85-fold) and protocadherin alpha subfamily C, 2 (Pcdhac2; 0.83-fold). Although both genes encode cell adhesion proteins, Spon2 is involved in the innate immune response, whereas Pcdhac2 affects nervous system development. Other nervous-system-related down-regulated genes included adenylate cyclase 8 (Adcy8), vasoactive intestinal peptide receptor 2 (Vipr2), ceramide synthase 2 (Cers2), ATPase, Na^+^/K^+^ transporting, beta 2 polypeptide (Atp1b), nuclear receptor subfamily 4 group A member 1 (Nr4a1), nuclear receptor subfamily 4 group A member 3 (Nr4a3), tropomodulin 2 (Tmod2), and endothelin receptor type A (Ednra).

**Figure 2:**
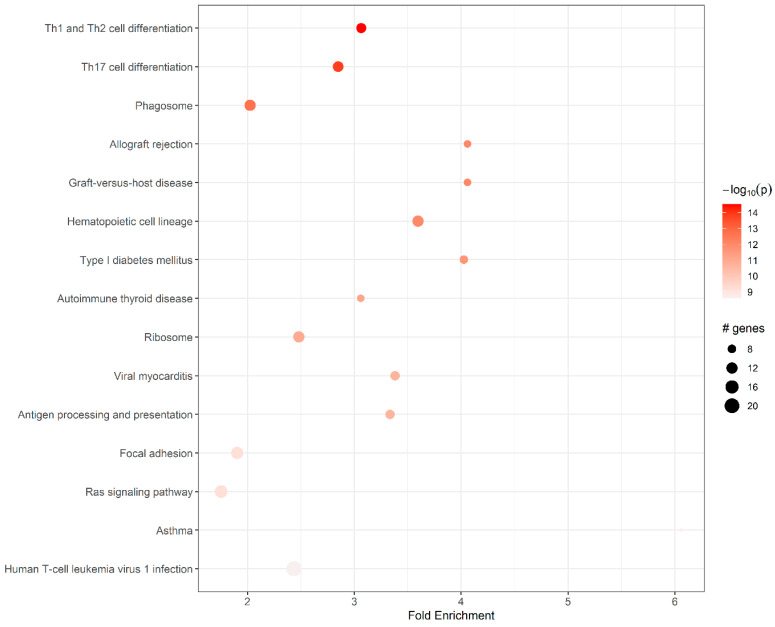
Top 15 enriched pathways based on DEGs (21 dpi) of IAV. Pathway analysis allowed the construction of a scatter plot of KEGG pathway enrichment analysis of DEGs after infection. The dot size indicates the number of genes in this pathway, and the intensity of red shows increasingly significant *p*-values.

KEGG enrichment analysis was further performed to identify the putative functions of the unique DEGs in these unique gene sets. The results are shown in Table 5 and Figure 2. The determined DEGs were mainly allocated to 15 pathways (Supplementary Table S1). The most significant fold enrichments were noted for immune-related pathways including asthma, antigen processing/presentation, hematopoietic cell lineage, allograft rejection, graft-versus-host disease, Th1/Th2 cell differentiation, and Th17 cell differentiation.

For the set of DEGs allocated to specific pathways, most up-regulated DEGs were related to six pathways, namely human T cell leukemia virus 1 infection, ribosome, hematopoietic cell lineage, phagosome, Th1/Th2 cell differentiation, and Th17 cell differentiation. Within the set of H1N1 uniquely up-regulated genes, human T cell leukemia virus 1 infection, ribosome, hematopoietic cell lineage, phagosome, Th1/Th2 cell differentiation, and Th17 cell differentiation were significantly enriched. The most uniquely down-regulated pathways in lungs after infection mainly involved focal adhesion and the Ras signaling pathway. One of the genes, Fos, was down-regulated in several pathways with fold enrichment ranging from 2.44 to 3.07 (Supplementary Table S1).

## 4. Discussion

The relationship between viral pathogenesis and host cell responses is a critical basis for infectious disease research because viral infections of the host cells prompt the expression of various genes that have a pivotal role in the initiation/development of infection [7]. Our study has identified the transcriptome profiles of infected and non-infected mouse lungs, revealing neural gene expression changes, plus increases in multiple immune- and inflammation-related pathways (e.g., B- and T-lymphocyte infiltration, innate immunity, immune-modulatory genes, cytokines/chemokines, interferon signaling, and leukocyte chemotaxis). We analyzed DEGs at 21 dpi (recovery stage) only because we intended to reveal the long-term potential effects/complications of IAV infection on the host [24,25]. In the case of severe acute respiratory syndrome coronavirus 2 (SARS-CoV-2) infection, these long-term potential effects/complications persisting or developing after an initial infection are called “long coronavirus disease 19 (COVID-19)” (or long COVID) [26,27,28,29]. Recovery from IAV infection was also evident in our results from the elevated expression of airway repair genes (especially keratins) and cellular proliferation genes (particularly those associated with the cell cycle). This was confirmed in our histopathological analysis by using H&E staining and immunostaining. These observations suggest that tissue repair processes continue to occur a long time after the acute early and intermediate phases of the host response to an influenza infection. Moreover, these processes might lead to lasting changes in the gene expression profiles within the lung, even after the infection has subsided [8,30].

Among 793 DEGs, 434 genes were up-regulated, and 358 genes were down-regulated. The most prominent molecular functions of up-regulated genes were related to the immune response and tissue repair, whereas a large proportion of down-regulated genes were associated with neural functions. This suggested alternations of both immune and neural activities/functions at the recovery stages of IAV infection. At 21 dpi, the up-regulated genes were related to the specific immune cells (e.g., T cells, B cells, and granulocyte/monocytes), inflammatory/immunomodulatory cytokines, and chemokine responses. For example, up-regulation of immunoglobulin heavy variable 14-2 (Ighv14-2) and Igkv5-48 indicated an increased humoral adaptive immunity, whereas up-regulation of granzyme K (GZMK) indicated an increased cell-mediated adaptive immunity. Our Transcriptomic results agreed with a few previously reported studies of IAV infection [8,31,32]. Some up-regulated genes at 21 dpi (e.g., Ltf, Cxcl9, Pglyrp1, Prg2, Cxcl13, Cxcl3, Ccl9, Ccl19, Krt5, Krt14, Krt15, Krt17, and Slpi) were also identified in a previous infection study by using H1N1(PR8) [8]. However, we found more up- and down-regulated genes compared with that study. In addition, changes in neural gene expression suggested potential neuroimmune interactions, neuroinflammation/neurotoxicity, and neuromodulation in the lungs (Table 2) [25,33].

Normally, the IAV mainly infects the respiratory system and causes respiratory illness (flu) [2]. However, under certain conditions (particularly severe ones), IAV can also affect the nervous system to variable degrees [34,35,36]. The involvement of the nervous system in IAV infections is not uncommon and can have serious consequences [34,35,36]. Neurological complications associated with influenza might result from direct viral invasion of the central nervous system (CNS) or indirectly through the body’s immune response and inflammation [25,34,35,36].

Influenza viruses can be classified into two main categories based on their ability to infect and affect the nervous system: neurotropic and non-neurotropic [25,35,37,38,39]. The vast majority of influenza infections are caused by non-neurotropic influenza viruses. Neurotropic influenza viruses are relatively rare, but when they do occur, they can lead to severe and potentially life-threatening neurological complications. Neurotropic influenza viruses have the ability to invade and infect the CNS, which includes the brain and spinal cord. These viruses can cross the blood–brain barrier and directly infect nerve cells, leading to neurological complications. The presence of the virus in the CNS can trigger inflammatory responses and damage to brain tissues, resulting in various neurological symptoms [25,37]. Neurotropic influenza viruses can cause conditions such as encephalitis and meningitis [25,37].

Non-neurotropic influenza viruses, on the other hand, do not have a strong affinity with the nervous system [38]. These viruses primarily infect and replicate in the respiratory system, causing typical flu-like symptoms such as cough, sore throat, fever, and body aches. Although they can cause severe respiratory illness, they do not usually invade the CNS or cause direct neurological complications [40]. However, the host immune response can lead to the production of proinflammatory/inflammatory cytokines (e.g., interleukin-6 (IL-6), interleukin-1α (IL-1α), and tumor necrosis factor-α (TNF-α)) and then affect cognition/emotional behaviors and other neurological functions [38,40].

Like many other internal organs, the lungs are innervated by sympathetic, parasympathetic, and sensory nervous systems that regulate the function of cells within the respiratory tract [33,41,42,43]. The neuroimmune interactions in the lungs might have essential roles in the host antiviral immune response [41,44].

The H1N1 virus used in our study is a non-neurotropic influenza virus [38,45]. A recent study has shown that neurotrophin signaling is impaired in CNS infection by the neurotropic influenza virus [46]. In our study, we observed the down-regulation of five genes (guanylate cyclase soluble subunit alpha-1 (Gucy1a), TEK receptor tyrosine kinase (Tek), solute carrier family 39 member 10 (Slc39a10), calmodulin-like protein 3 (Calml3), mitogen-activated protein kinase kinase kinase 1 (Map3k1)) related to neurotrophin signaling. By using RT-PCR, we observed the down-regulation of BDNF in mouse lungs during the infection with IAV. Since neurotrophins are critical modulators of neuroinflammation, blood–brain barrier permeability, apoptosis, learning/memory capacity, and neurite regeneration [19,47,48], the down-regulation of these genes might contribute to the neurological damage and complications that can occur after IAV infection. For example, BDNF has a negative regulatory role in the resolution of neuroinflammation, and a highly inflammatory condition might reduce BDNF expression [49].

We also observed the down-regulation of many other genes at 21 dpi. For example, seven genes (Abl interactor 3 binding protein (Abi3bp), Vinculin (Vcl), CDC42 effector protein 1 (Cdc42ep1), solute carrier family 9 member A3 regulator 2 (Slc9a3r2), CLIP-associated protein 2 (Clasp2), actin binding LIM protein family member 3 (Ablim3), and Fermitin family homolog 2 (Fermt2)) related to actin organization were down-regulated. Since abnormal regulation of the actin cytoskeleton might contribute to several pathological conditions primarily affecting the nervous system [50], the down-regulation of these genes might result in neurological complications in IAV infection. In addition, the down-regulation of actin-organization-related genes caused by virus infection might also lead to a delayed airway epithelium wound repair process (shown by epithelial cell necrosis; Supplementary Figure S2), as reported in another infection study in which respiratory syncytial virus was used [51].

In the search for neuroinflammation-related DEGs, two neural genes (calcitonin gene-related peptide-β (Calcb) and potassium voltage-gated channel subfamily A member 6 (Kcna6)) were notably up-regulated. Calcb is primarily expressed in the central and peripheral nervous systems, where it acts as a potent vasodilator and neuromodulator. It plays a significant role in the regulation of vascular tone, pain transmission, and neurogenic inflammation [52,53,54]. In addition, Calcb is an essential neurotransmitter in the neuroimmune axis and may be involved in the cytokine storm and pulmonary pathophysiology of COVID-19 [55,56,57].

Kcna6 channels are predominantly expressed in the nervous system, where they contribute to the shaping of the action potential and neuronal excitability. Dysregulation of these channels has been implicated in various neurological disorders, making them a potential target for therapeutic interventions aimed at modulating neural activities [58]. In addition, Kcna6 may be used as a target for treating COVID-19 since it can facilitate the entry of SARS-CoV-2 into neurons [59].

We also observed that seven neurogenic genes, Adcy8, Vipr2, Cers2, ATP1B2, Nr4a1, Nr4a3, Tmod2, and Ednra, were up-regulated by 21 dpi of IAV (Table 4).

Adcy8 is an enzyme that catalyzes the production of cyclic adenosine monophosphate (cAMP), a critical second messenger in cellular signaling pathways. In the nervous system, Adcy8 has been linked to various neurological functions [60]. For example, it plays a role in modulating synaptic plasticity, which underlies learning and memory processes. Adcy8 also influences neurotransmitter release, thereby affecting synaptic communication and neuronal excitability [61]. Dysregulation of Adcy8 has been associated with neurological disorders such as epilepsy, mood disorders, and neurodegenerative diseases, suggesting its potential as a target for therapeutic interventions. The down-regulation of Adcy8 observed in our study indicates the potential alternation of PNS functions by viral infection and the effect of lung innervation in the host response to IAV infection.

Vipr2 is a G protein-coupled receptor that binds to the neuropeptide vasoactive intestinal peptide (VIP). It is widely expressed in various tissues, including the nervous system, gastrointestinal tract, respiratory system, and immune system [62]. In the CNS, Vipr2 is involved in regulating several physiological processes, including neurotransmission, synaptic plasticity, and neuroprotection. In the lungs, VIP is one of the most important noradrenergic noncholinergic inhibitory transmitters involved in airway tone control, vascular relaxation, and mucus secretion [63]. In the context of innate immunity, VIP acts as an inhibitor, effectively reducing the production of inflammatory cytokines/chemokines in macrophages, DCs, and microglia. Moreover, VIP plays a role in down-regulating the expression of co-stimulatory molecules on antigen-presenting cells, ultimately leading to a decrease in the activation of antigen-specific CD4^+^ T cells [64]. Therefore, the down-regulation of Vipr2 indicates the potential impairment of the immune response inside the lungs.

Nr4a is a group of transcription factors that play crucial roles in various physiological processes [65]. Nr4a members include three related proteins: Nr4a1 (Nur77 or TR3), Nr4a2 (Nurr1), and Nr4a3 (Nor-1). These receptors are unique because they can regulate gene expression both as transcriptional activators and repressors, influencing diverse biological pathways. NR4a proteins are involved in the regulation of cell proliferation, apoptosis, metabolism, inflammation, and immune responses, making them critical players in maintaining cellular homeostasis. Nr4a1 can act as a microglia variable resistor and can be used as a therapeutic target in autoimmune CNS inflammation [66].

In total, 15 significantly enriched KEGG pathways were identified as DEGs (Figure 2). During viral infections, the differentiation of T helper (Th) cells is crucial. Th1 cells promote cellular immune responses, activating cytotoxic T cells/macrophages to eliminate infected cells, whereas Th2 cells stimulate humoral immunity, promoting B cell activation and antibody production to combat extracellular pathogens [67]. Th17 cells play a crucial role in the immune response to viral infections by producing proinflammatory cytokines, such as interleukin-17 (IL-17), which help to recruit and activate immune cells to combat the virus [68,69]. They contribute to the defense against certain extracellular viruses and aid in the clearance of infected cells. However, dysregulated Th17 responses can lead to excessive inflammation and tissue damage, highlighting the importance of balanced Th17 activation during viral infections [68,69]. For example, studies have suggested that Th17 cells play a crucial role in COVID-19 pathogenesis, not only by activating the cytokine signaling/cascade but also by stimulating Th2 responses, inhibiting Th1 differentiation, and suppressing regulatory T cells (Tregs) [70].

Phagosomes are specialized compartments lying within immune cells (e.g., macrophages and DCs) that engulf and internalize viral particles or infected cells [71]. Once inside the phagosome, the viral material is degraded through a series of enzymatic processes, enabling the presentation of viral antigens to other immune cells, and initiating a coordinated antiviral immune response. Therefore, the up-regulation of the phagosome pathway genes indicates increased innate immunity (phagocytosis of virus by DCs and macrophages) and adaptive immunity (antigen presentation) after IAV infection [71]. The increase in antigen presentation can also be observed in the up-regulation of the antigen processing/presentation pathway seen in this study.

During viral infections, the Ras signaling pathway can be influenced by certain viruses to promote their replication and to enable them to evade the host immune response [72]. Some viruses, such as IAVs, can activate the Ras pathway to enhance cell proliferation and survival, benefitting their replication and spread [73]. The down-regulation of the Ras signaling pathway observed in this study might improve the host response to the viral infection (e.g., up-regulation of the type I interferon antiviral response [74]). The targeting of the Ras signaling pathway in viral infections has been explored as a potential therapeutic approach for disrupting viral replication and limiting viral-induced cellular changes [73].

Although our results have demonstrated that these signaling pathways are closely related to IAV infection, further investigation of these enriched signaling pathways is needed in order to gain a better understanding of the long-term effects/complications of IAV infection on the lungs and other organs (e.g., brain, spinal cord, and liver).

One limitation of our study is that we have mainly checked the transcriptional changes at 21 dpi. Examination of the transcriptional changes for an even longer time period (e.g., 60 dpi or even longer), as described in some previous studies [25,30,38], is of interest. Furthermore, a comparison of H1N1 (non-neurotropic) with other neurotropic IAVs (e.g., H7N7) by using RNA-seq will be meaningful with regard to their effects on transcriptomic profiles/changes [25,30,38].

## 5. Conclusions

RNA-seq enables an accurate transcriptomic analysis of gene expression and of relevant enriched pathways during H1N1 infection. Our studies have revealed many DEGs that are associated with non-specific (innate) and specific (humoral and cellular-mediated) immune responses, mucosal immunity, inflammatory responses, airway repair, modulated immune responses, and neural responses. Although further molecular/functional studies need to be performed for these DEGs, our results might facilitate the understanding of the host response (from innate immunity to adaptive immunity) and neuroimmune interactions in the lungs at the recovery stage of IAV infection. Ultimately, these genes might have potential uses as mechanistic/diagnostic biomarkers and be possible targets for anti-IAV therapies in the future.

## Figures and Tables

**Table 1:** Top 100 up-regulated DEGs in the lung tissue of IAV-infected mice (21 dpi).

B Cell Infiltration/Plasma Cells	T Cell Infiltration	Airway Repair
Igh; Igk; Cd79a; Cd22; Mzb1; Rgs13; Tnfrsf17; Tnfrsf13c; Spib; Pou2af1; Fcamr; Aicda; Jchain; Cd52	Gzmk; Cxcr3; Tigit; Thy1; Cd4; Cd3; Izumo1r; Pbk; Cd52	Krt5; Krt14; Krt15; Krt17; Mef2b; Slpi; Tff2
Leukocyte Chemotaxis	Inflammation and Edema	Immune Modulation
Ccl20; Oit1; Ccl8; Cxcl9; Cxcl10; Cxcl13	Itln1; Ubd; Aqp3; Agr2; Clca1; Il9r	Pla2g2d; Tigit; Calcb; Sectm1b; Il21r; Akr1c18
Cell Proliferation	Innate Immunity	Platelets/Platelet Activation
Cdca5; Ccna1; Ccna2; Ccnb1; Plk1; Ska1; Cdk1; Cdkn3; Cdca3; Cdca8; Cdc20; Cenpm; Cep55; Mxd3; Melk; Top2a	Ltf; Dmbt1; Reg3g; Pglyrp1; Padi1; Bpifa1; Gp2	Alox12
Granulocyte/Monocyte Infiltration	Hypoxia	Smooth Muscle Activation
Prg2; Ms4a7; Cd177	Dmbt1	Stra6; Adh7; Dnase1l3; Birc5
Mesothelium	MHC	Cell Signaling and Apoptosis
Msln	H2-EB2; H2-M2	Stra6; Adh7; Dnase1l3; Birc5
Complement	Neuronal	
C1qa; C1qb	Calcb; Kcna6	

**Table 2:** Top 100 down-regulated DEGs in the lung tissue of IAV-infected mice (21 dpi).

Innate Immune Response	Specific Immune Response	Inflammatory Response
Spon2	Slc39a10; S1pr1	Abi3bp; Adcy8; Mylk; Tspan18
Anti-inflammation	Neuroinflammation	Brain Development
Tek	Adcy8	Pcdh18; Dnm1l; Tmod2; Adcy8
Gene Expression/Transcription Regulation	Cell Division	Neurotrophin Signaling Pathway
Rnf187; Nox4; Zeb1; Aff1; Ier2; Tfcp2l1; Zeb2; Tfdp2; Klf11; Sox7	Pard6g; Usp39; Specc1l	Gucy1a1; Tek; Slc39a10; Calml3; Map3k1
Cell Death (i.e., Apoptosis)	cAMP Synthesis	Actin Organization
Peg3; Mylk; Dnm1l; Tnfrsf19	Adcy8	Abi3bp; Vcl; Cdc42ep1; Slc9a3r2; Clasp2; Ablim3; Fermt2
Cell Adhesion	Neuronal	Growth Factor Activity
Spon2; Vcl; Ptprm	Ier2; Sema6d; Zdhhc3; Map6; Zeb1; Gm38399; Scn3a; Peg3; Atp7a; Tmod2; Abcd2; Map3k11; Vcl; Adcy8; Luzp1; Amph; Sema6d; mylk	Ier2; Reps2; Cpm; Rnf187; Ogn; Flt4; Tek

**Table 3:** Top 20 up-regulated DEGs in the lungs of IAV-infected mice (21 dpi) compared with control mice.

Gene	Full Name	Molecular Functions	Log2 FC	Fold Up	*p*-Value
Ighv14-2	Immunoglobulin heavy variable 14-2	Humoral immune response	5.41	42.56	4.70 × 10^−9^
AICDA	Activation-induced cytidine deaminase	Somatic hypermutation and antibody class switching	5.40	42.33	3.89 × 10^−7^
GZMK	Granzyme K	Cytolytic granules of cytotoxic T lymphocytes and natural killer cells; Inhibitor of influenza virus replication	3.92	15.08	0.000107
TIGIT	T cell immunoreceptor with Ig and ITIM domains	Induces IL-10; Suppresses T cell activation by generating immunoregulatory dendritic cells (DCs)	3.20	9.19	4.57 × 10^−5^
PRG2	Proteoglycan 2 (bone marrow)	Induces non-cytolytic histamine release; Eosinophil major basic protein	4.70	26.03	1.80 × 10^−6^
KRT14	Keratin 14	Airway repair via epithelial cell differentiation	6.47	88.94	1.24 × 10^−5^
ALOX12E	Arachidonate lipoxygenase, epidermal	Prothrombotic/inflammatory response during influenza virus infection; Regulates gene expression	7.35	162.72	1.29 × 10^−6^
ITLN1	Intelectin 1	Airway inflammation in mucsa	5.35	40.87	1.95 × 10^−8^
LTF	Lactotransferrin	Negatively regulates viral processes and inhibits viral genome replication	4.77	27.26	4.35 × 10^−6^
DMBT1	Deleted in malignant brain tumors 1	Mucosal immune defense, epithelial differentiation, and tumor suppression	4.43	21.55	2.83 × 10^−5^
CCL20	Chemokine (C-C motif) ligand 20	Induces strong chemotactic for lymphocytes	4.49	22.52	8.50 × 10^−8^
BPIFA1	BPI fold-containing family A member 1	Neutrophil recruitment and interferon signaling; Inhibits viral proliferation; Immune response in upper airway	3.92	15.17	0.00039
Igkv5-48	Immunoglobulin kappa variable 5-48	Humoral immune response	6.02	64.83	1.11 × 10^−9^
MEF2B	Myocyte enhancer factor 2B	Smooth muscle-specific and/or growth-factor-related transcription	4.48	22.36	1.37 × 10^−6^
CD177	CD177 antigen	Mediates activation of TNF-α primed neutrophils	1.87	3.66	0.000317
CALCB	Calcitonin-related polypeptide, beta	Neuroimmune communicator to regulate lymphocytes; Suppresses appetite	3.41	10.67	9.85 × 10^−5^
KCNA6	Potassium voltage-gated channel, shaker-related, subfamily A, member 6	Provide instructions for making voltage -gated potassium channels; Regulates neurotransmitter release, heart rate, insulin secretion, neuronal excitability, epithelial electrolyte transport, smooth muscle contraction, and cell volume	1.69	3.22	0.00022
MSLN	Mesothelin	Cellular adhesion	3.41	10.66	1.92 × 10^−6^
PLA2G2D	Phospholipase A2, group IID	Anti-inflammatory and immunosuppressive functions; Generates lipid mediators for pathogen clearance	3.11	8.63	1.38 × 10^−5^
H2-EB2	Histocompatibility 2, class II antigen E beta2	Adaptive immune response; Antigen processing; Presentation of peptide via MHCII	2.92	7.55	1.36 × 10^−5^

**Table 4:** Top 20 down-regulated DEGs in the lungs of IAV-infected mice (21 dpi) compared with control mice.

Gene	Full Name	Molecular Functions	Log2 FC	Fold Up	*p*-Value
Abi3bp	ABI family member 3 binding protein	Actin filament and collagen binding; Inflammatory response	−1.19	0.44	7.10 × 10^−7^
Vcl	Vinculin	Cell-matrix and cell–cell adhesion	−1.49	0.36	7.62 × 10^−7^
Adcy8	Adenylate cyclase 8	Neuroinflammatory response and brain functions such as memory; cAMP signaling activation	−1.35	0.39	2.62 × 10^−6^
Vipr2	Vasoactive intestinal peptide receptor 2	Receptor for VIP; Water and ion flux in lungs and intestinal epithelia	−1.45	0.37	9.96 × 10^−6^
Pcdhac2	Protocadherin alpha subfamily C, 2	Nervous system development; calcium-dependent cell-adhesion protein	−2.57	0.17	1.03 × 10^−5^
Spon2	Spondin 2	Cell adhesion and innate immune response	−2.74	0.15	2.34 × 10^−13^
Elmo2	Engulfment and cell motility 2	Cytoskeletal rearrangements for phagocytosis of apoptotic cells	−0.90	0.54	1.58 × 10^−5^
Eng	Endoglin	Regulates angiogenesis and CNS vasculogenesis	−1.30	0.40	2.78 × 10^−5^
Cers2	Ceramide synthase 2	Negative regulation of Schwann cell migration and proliferation in axon regeneration	−0.82	0.56	0.000188
Atp1b2	ATPase, Na ^+^ /K ^+^ transporting, beta 2 polypeptide	Mediates cell adhesion of neurons and astrocytes; Catalyzes the hydrolysis of ATP	−0.86	0.55	0.000168
Hsph1	Heat shock protein 1 105 kDa	Positive regulation of natural killer T cell activation	−1.12	0.46	0.00017
Nr4a1	Nuclear receptor subfamily 4 group A member 1	Neurotransmitter secretion; Cell cycle mediation, inflammation, and apoptosis	−1.01	0.50	0.000215
Nr4a3	Nuclear receptor subfamily 4 group A member 3	Inflammatory response; Mediates survival of neurons and smooth muscle cells	−1.17	0.44	0.000206
Tmod2	Tropomodulin 2	Neuron–neuron synaptic transmission; Learning or memory	−0.81	0.57	0.000123
Ednra	Endothelin receptor type A	Development of enteric nervous system and neural crest cells	−0.78	0.58	4.06 × 10^−5^
S1pr1	Sphingosine 1- phosphate receptor 1	Immune response; Transport of mature T cells from the thymus into the blood and peripheral lymphoid organs	−1.01	0.50	6.91 × 10^−5^
Mylk	Myosin light chain kinase	Inflammatory response; Regulating the actin–myosin interaction of smooth muscle	−0.83	0.56	8.89 × 10^−5^
Crtc3	CREB-regulated transcription coactivator 3	cAMP response element building	−1.07	0.48	9.65 × 10^−5^
Slc39a10	Solute carrier family 39, member 10	B cell proliferation and B cell receptor signaling pathway	−0.82	0.57	0.000119
Usp39	Ubiquitin-specific peptidase 39	Cell cycle and cell division; Pre-mRNA splicing; NF-κB-mediated inflammatory responses	−1.12	0.46	0.000153

**Table 5:** Top 15 enriched pathways based on DEGs (21 dpi) of IAV.

ID	Pathway	Fold En.	Up-Regulated Genes	Down-Regulated Genes
mmu04658	Th1 and Th2 cell differentiation	3.07	H2-Eb1, H2-DMa, H2-Oa, H2-Ob, H2-Eb2, Cd4, Cd3g, Cd3d, Lat	Fos
mmu04659	Th17 cell differentiation	2.85	Il21r, H2-Eb1, H2-DMa, H2-Oa, H2-Ob, H2-Eb2, Cd4, Cd3g, Cd3d, Lat	Fos
mmu04145	Phagosome	2.03	H2-M2, H2-Q6, H2-Eb1, H2-DMa, H2-Oa, H2-Ob, H2-Eb2, Tap2, Cd14	Sec61a1, Thbs1, Cd209b
mmu05330	Allograft rejection	4.06	H2-M2, H2-Q6, H2-Eb1, H2-DMa, H2-Oa, H2-Ob, H2-Eb2	
mmu05332	Graft-versus-host disease	4.06	H2-Eb1, H2-DMa, H2-Oa, H2-Ob, H2-Eb2, H2-M2, H2-Q6	
mmu04640	Hematopoietic cell lineage	3.60	H2-Eb1, H2-DMa, H2-Oa, H2-Ob, H2-Eb2, Cd4, Cd3d, Cd3g, Cd22, Cd14,	Kit
mmu04940	Type I diabetes mellitus	4.03	H2-Eb1, H2-DMa, H2-Oa, H2-Ob, H2-Eb2, H2-M2, H2-Q6	Ica1
mmu05320	Autoimmune thyroid disease	3.06	H2-Eb1, H2-DMa, H2-Oa, H2-Ob, H2-Eb2, H2-M2, H2-Q6	
mmu03010	Ribosome	2.48	Rps7, Rps8, Rps10, Rps20, Rps23, Rps27a, Rpsa, Rpl11, Rpl22, Rpl22l1, Rpl30, Rpl35a	
mmu05416	Viral myocarditis	3.38	H2-Eb1, H2-DMa, H2-Oa, H2-Ob, H2-Eb2, H2-M2, H2-Q6	Cav1, Icam1
mmu04612	Antigen processing and presentation	3.33	H2-M2, H2-Q6, Tap2, H2- Eb1, H2-DMa, H2-Oa, H2- Ob, H2-Eb2, Cd4	
mmu04510	Focal adhesion	1.90	Prkcb	
mmu04014	Ras signaling pathway	1.75	Efna5, Lat, Calml3, Pla2g5, Pla2g2d, Prkcb	
mmu05310	Asthma	6.06	H2-Eb1, H2-DMa, H2-Oa, H2-Ob, H2-Eb2	
mmu05166	Human T cell leukemia virus 1 infection	2.44	Cd4, H2-M2, H2-Q6, Mad2l1, Cdc20, Ccnb2, Ccna2, Ccna1, Map3k1, Cd3d, Cd3g, Tnfrsf13c, H2-Eb1, H2-DMa, H2-Oa, H2-Ob, H2-Eb2	

## Data Availability

All data are included in the article and its Supplementary Materials.

## Supplementary Materials

The following supporting information can be downloaded at: https://www.mdpi.com/article/10.3390/v15112198/s1. Figure S1: Average percentage changes in body weight of control mice (*n* = 3) and infected mice with human IAV (H1N1 PR8; Infectious dose: 20 TCID_50_; *n* = 5); Figure S2: H&E staining of mouse lung cryosections (10 µm) showing the lung pathology at 21 dpi with human IAV (H1N1 PR8; Infectious dose: 20 TCID_50_) [75]; Figure S3: Immunofluorescence staining of mouse lung cryosections (20 µm) to examine lung histopathology at 21 dpi with human IAV (H1N1 PR8; infectious dose: 20 TCID_50_) [76]; Figure S4: Total reads of experimental samples of mice lungs at 21 dpi with human IAV (H1N1 PR8; infectious dose: 20 TCID_50_) showing good quality and consistency across all samples; Figure S5: The 792 DEGs (434 up-regulated and 358 down-regulated) identified at 21 dpi with human IAV (H1N1 PR8; infectious dose: 20 TCID_50_); Figure S6: Relative quantification of the BDNF, GFAP, and HA gene expression in lungs, thymus, and spleen of control mice and infected mice at 3, 7, 14, and 21 dpi with human IAV (H1N1 PR8; infectious dose: 20 TCID_50_) via RT-PCR [77]; Table S1: Significant molecular pathways determined in lung tissue of infected vs. control mice with reference to KEGG.
