# Supplementary material for: Transcriptomic Profiling of Influenza A Virus-Infected Mouse Lung at Recovery Stage Using RNA Sequencing
Source: Viruses. 2023 Oct 31;15(11):2198. doi: 10.3390/v15112198 (PMC10675624; doi:10.3390/v15112198)
Supplement: Supplementary file 1 [file viruses-15-02198-s001.zip › viruses-2574958-supplementary.pdf]

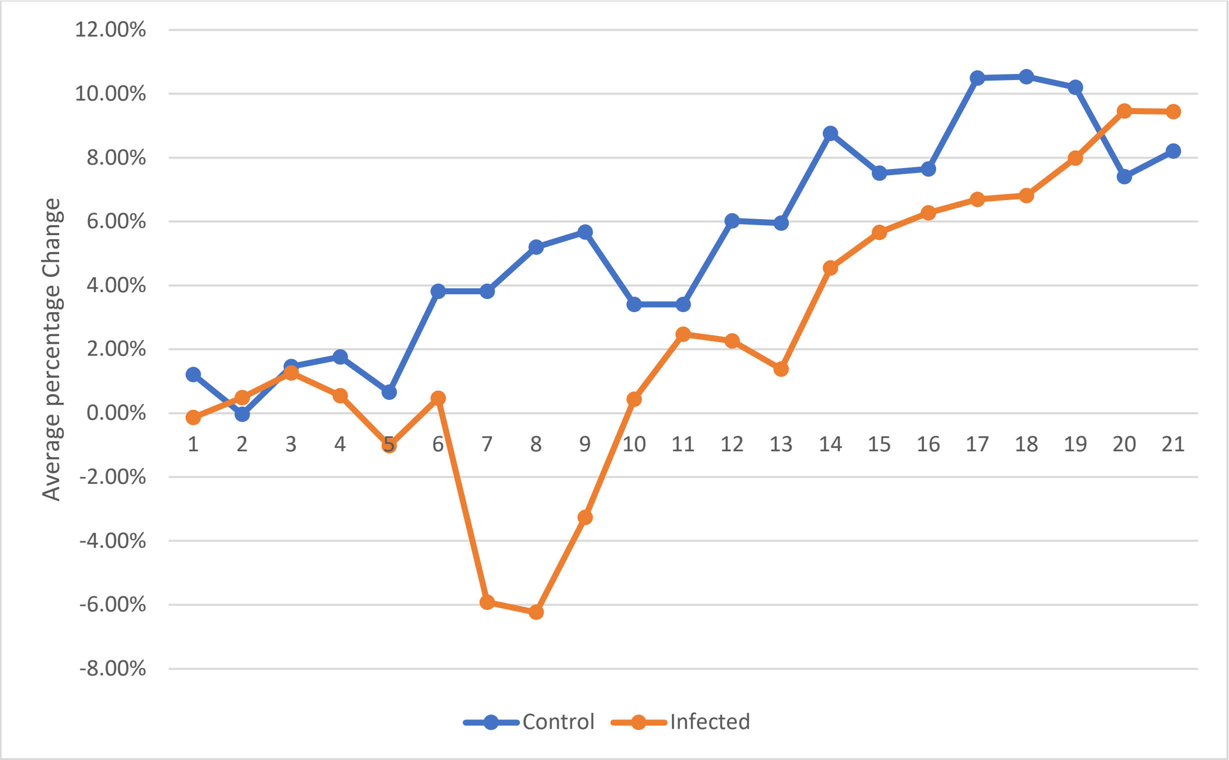

Supplementary Figure S1. Average percentage changes in body weight of control mice (*n*=3) and infected mice with human IAV (H1N1 PR8; Infectious dose: 20 $TCID_{50}$; *n*=5).

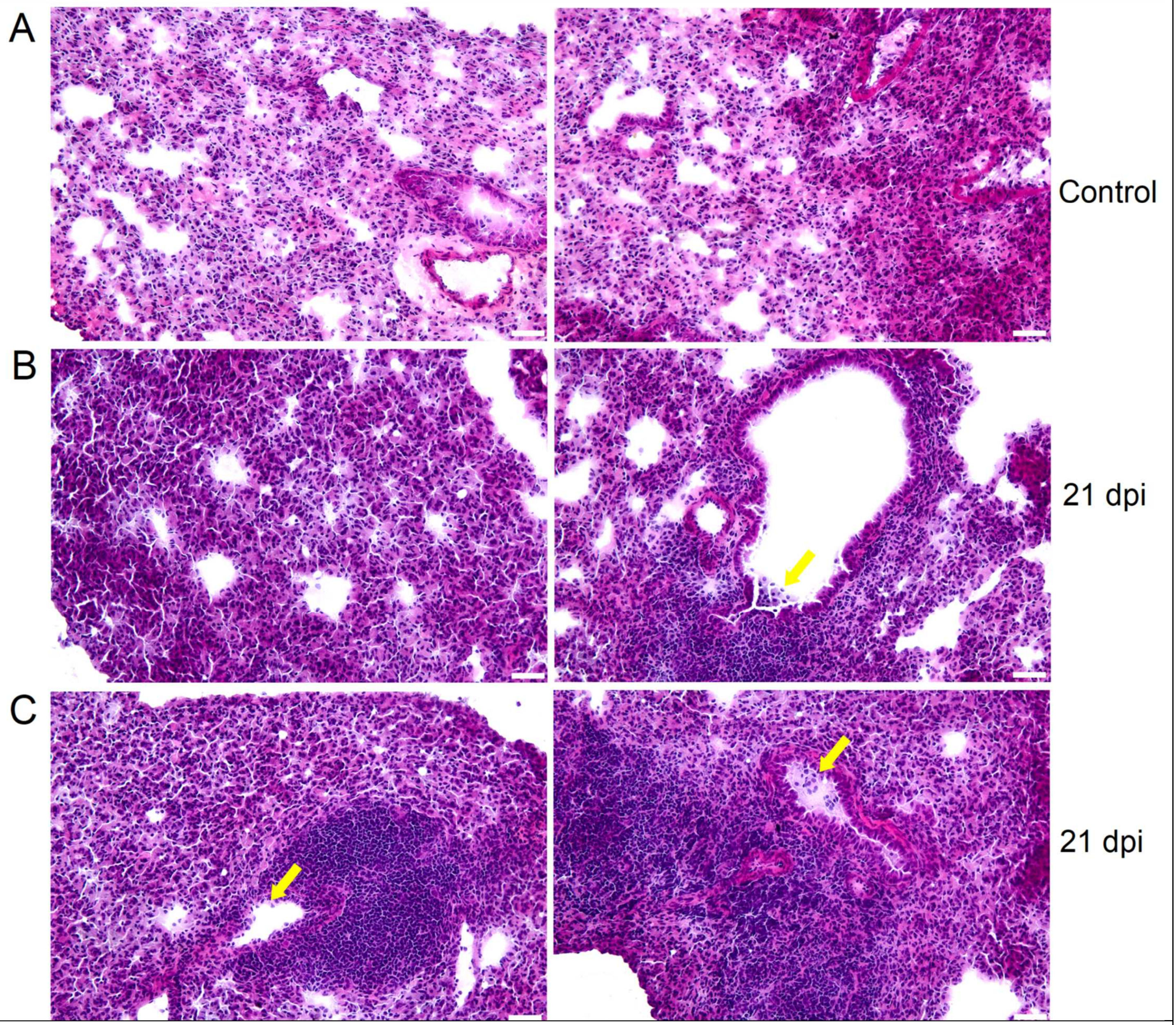

Supplementary Figure S2. H&E staining of mouse lung cryosections (10 µm) showing the lung pathology at 21 dpi with human IAV (H1N1 PR8; Infectious dose: 20 $TCID_{50}$). (A) In the control group, the tissue histology is normal. *n*=3. (B-C) Multifocal moderate broncho-interstitial infiltrate and inflammation are present inside the lungs of mice at 21 dpi. The yellow arrows indicate the epithelial cell necrosis inside the airway. *n*=5. Objective lens: 20×; Scale bar: 50 µm.

Materials and Methods

Cryosections were prepared and stained as previously described [74].

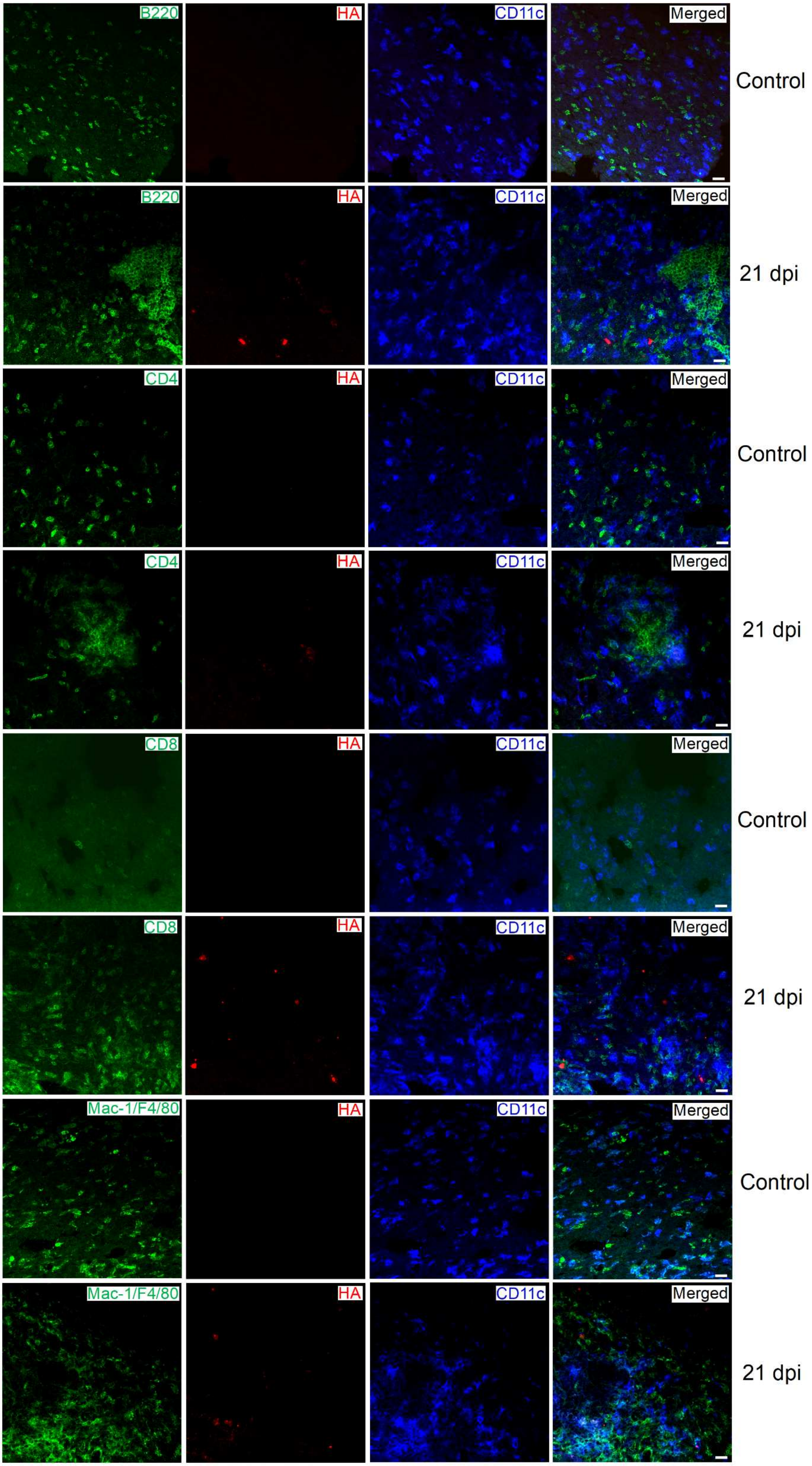

B220
HA
CD11c
Merged
Control
21 dpi
CD4
CD8
Mac-1/F4/80

Supplementary Figure S3. Immunofluorescence staining of mouse lung cryosections (20 µm) to examine lung histopathology at 21 dpi with human IAV (H1N1 PR8; infectious dose: 20 $TCID_{50}$). Antibodies against B220/CD4/CD8a/[Mac-1/F4/80] (green), hemagglutinin (red), and CD11c (blue) label B cells/T helper cells/cytotoxic T cells/macrophages, H1N1 virus, and DCs, respectively. HA: hemagglutinin. Each image is a maximal intensity projection of a Z-stack. Stack size: 8.0 µm; optical slice interval: 0.50 µm; Objective lens: 40×; Scale bar: 20 µm.

Materials and Methods

Cryosections were prepared and stained as previously described [75].

Specificities and sources of primary and secondary antibodies

**Target (alternative name) [cat. no.]**	**Conjugate**	**Species and isotype**	**Main cells labeled**	**Dilution**	**Company**
CD11c [60002]	—	Armenian hamster monoclonal IgG	Dendritic cell	1:500	STEMCELL Technologies (Tullamarine, Australia)
B220 (CD45R) [103202]	—	Rat monoclonal IgG	B cell	1:300	Australian Biosearch (Karrinyup, Australia)
CD4 [100506]	—	Rat monoclonal IgG	T helper cells	1:300	Australian Biosearch
CD8a [100802]	—	Rat monoclonal IgG	Cytotoxic T cells	1:300	Australian Biosearch
Mac1(CD11b) [101202]	—	Rat monoclonal IgG	Macrophages	1:300	Australian Biosearch
F4/80 [123102]	—	Rat monoclonal IgG	Macrophages	1:300	Australian Biosearch
Influenza A PR8/34 H1N1 Hemagglutinin [11684-R107]	—	Rabbit monoclonal IgG	H1N1 virus	1:500	Sino Biological (Beijing, China)
Rabbit IgG H&L [A-21428]	Alexa Fluor® 555	Goat polyclonal	—	1:1000	Thermo Fisher Scientific (Waltham, MA, USA)
Armenian Hamster IgG H&L [ab173004]	Alexa Fluor® 647	Goat polyclonal	—	1:1000	Abcam Australia (Melbourne, Australia)
Rat IgG H&L [ab150157]	Alexa Fluor® 488	Goat polyclonal	—	1:1000	Abcam Australia

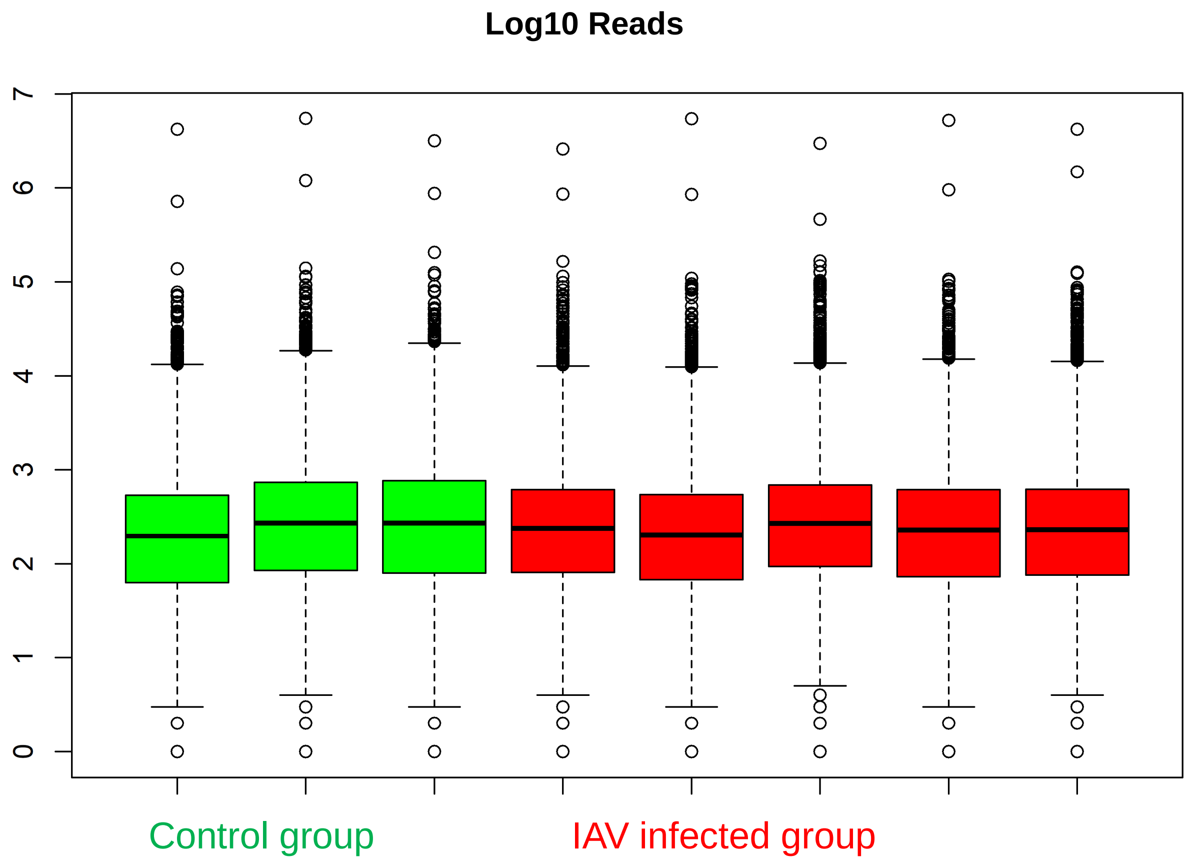

Supplementary Figure S4. Total reads of experimental samples of mice lungs at 21 dpi with human IAV (H1N1 PR8; infectious dose: 20 $TCID_{50}$) showing good quality and consistency across all samples. Control group: *n*=3; Infected group: *n*=5.

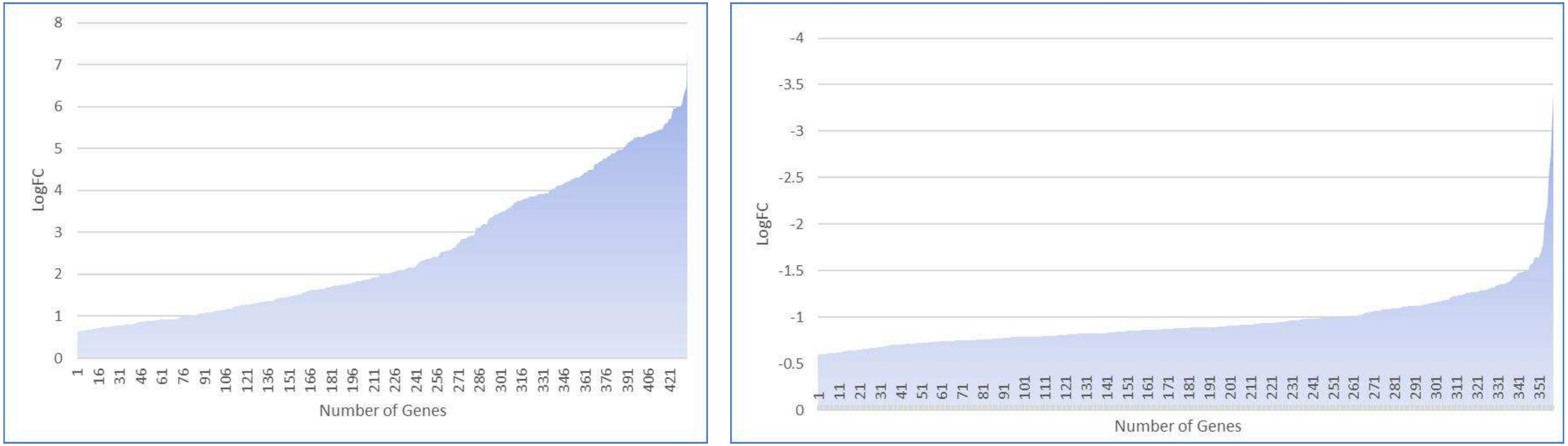

Supplementary Figure S5. The 792 DEGs (434 up-regulated and 358 down-regulated) identified at 21 dpi with human IAV (H1N1 PR8; infectious dose: 20 $TCID_{50}$). Control group: *n*=3; Infected group: *n*=5.

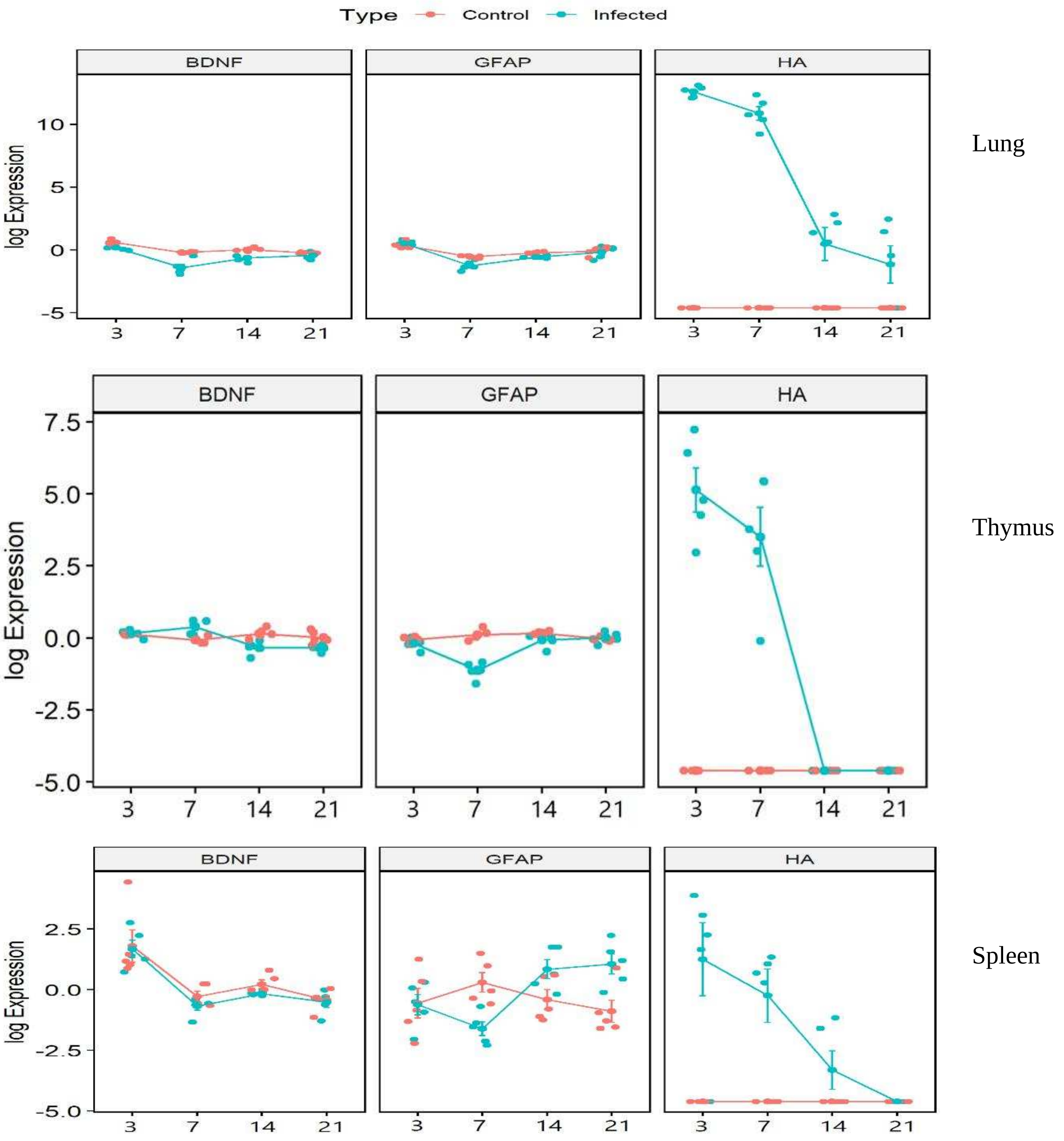

Supplementary Figure S6. Relative quantification of the BDNF, GFAP, and HA gene expression in lungs, thymus, and spleen of control mice and infected mice at 3, 7, 14, and 21 dpi with human IAV (H1N1 PR8; infectious does: 20 $TCID_{50}$) via RT-PCR. *n*=3.

Materials and Methods

RT-PCR was performed as previously described [76].

The sequences and sources of primers used in the RT-PCR

**Gene**	**Forward primer**	**Reverse primer**	**Amplicon length**
β-actin	CTGGGACGACATGGAGAAGATC	GTCTCAAACATGATCTGGGTCATC	144 bp
GFAP	ACCGCATCACCATTCCTGTAC	TGGCCTTCTGACACGGATTT	85 bp
BDNF	GTCTGACGACGACATCACTGG	AGAGGAGGCTCCAAAGGCACT	93 bp
HA-IAV	TATTTGGAGCCATTGCCGGT	GATCCGCTGCATAGCCTGAT	108 bp

Supplementary Table S1 Significant molecular pathways determined in lung tissue of infected vs. control mice with reference to KEGG

	**ID**	**Pathway**	**Fold En.**	**Up-regulated genes**	**Down-regulated genes**
1	mmu04658	Th1 and Th2 cell differentiation	3.07	H2-Eb1, H2-DMa, H2-Oa, H2-Ob, H2-Eb2, Cd4, Cd3g, Cd3d, Lat	Fos
2	mmu04659	Th17 cell differentiation	2.85	Il21r, H2-Eb1, H2-DMa, H2-Oa, H2-Ob, H2-Eb2, Cd4, Cd3g, Cd3d, Lat	Fos
3	mmu04145	Phagosome	2.03	H2-M2, H2-Q6, H2-Eb1, H2-DMa, H2-Oa, H2-Ob, H2-Eb2, Tap2, Cd14	Sec61a1, Thbs1, Cd209b
4	mmu05330	Allograft rejection	4.06	H2-M2, H2-Q6, H2-Eb1, H2-DMa, H2-Oa, H2-Ob, H2-Eb2	
5	mmu05332	Graft-versus-host disease	4.06	H2-Eb1, H2-DMa, H2-Oa, H2-Ob, H2-Eb2, H2-M2, H2-Q6	
6	mmu04640	Hematopoietic cell lineage	3.60	H2-Eb1, H2-DMa, H2-Oa, H2-Ob, H2-Eb2, Cd4, Cd3d, Cd3g, Cd22, Cd14,	Kit
7	mmu04940	Type I diabetes mellitus	4.03	H2-Eb1, H2-DMa, H2-Oa, H2-Ob, H2-Eb2, H2-M2, H2-Q6	Ica1
8	mmu05320	Autoimmune thyroid disease	3.06	H2-Eb1, H2-DMa, H2-Oa, H2-Ob, H2-Eb2, H2-M2, H2-Q6	
9	mmu03010	Ribosome	2.48	Rps7, Rps8, Rps10, Rps20, Rps23, Rps27a, Rpsa, Rpl11, Rpl22, Rpl22l1, Rpl30, Rpl35a	
10	mmu05416	Viral myocarditis	3.38	H2-Eb1, H2-DMa, H2-Oa, H2-Ob, H2-Eb2, H2-M2, H2-Q6	Cav1, Icam1
11	mmu04612	Antigen processing and presentation	3.33	H2-M2, H2-Q6, Tap2, H2-Eb1, H2-DMa, H2-Oa, H2-Ob, H2-Eb2, Cd4	
12	mmu04510	Focal adhesion	1.90	Prkcb	
13	mmu04014	Ras signaling pathway	1.75	Efna5, Lat, Calml3, Pla2g5, Pla2g2d, Prkcb	
14	mmu05310	Asthma	6.06	H2-Eb1, H2-DMa, H2-Oa, H2-Ob, H2-Eb2	
15	mmu05166	Human T-cell leukemia virus 1 infection	2.44	Cd4, H2-M2, H2-Q6, Mad2l1, Cdc20, Ccnb2, Ccna2, Ccna1, Map3k1, Cd3d, Cd3g, Tnfrsf13c, H2-Eb1, H2-DMa, H2-Oa, H2-Ob, H2-Eb2	
